# Effectiveness of platelet concentrates, corticotomy, and piezocision as adjuncts for accelerating maxillary canine retraction: a systematic review of randomized controlled trials

**DOI:** 10.3389/fdmed.2026.1920750

**Published:** 2026-10-06

**Authors:** Arjun KV, Anmol Sharma, Azhar Mohammed, M. S. Ravi, Sunidhi Kamboj, Naveen Pulikkottil Sunny

**Affiliations:** Department of Orthodontics and Dentofacial Orthopaedics, Nitte (Deemed to be University), AB Shetty Memorial Institute of Dental Sciences (ABSMIDS), Mangalore, India

**Keywords:** accelerated orthodontics, canine retraction, corticotomy, piezocision, platelet-rich fibrin, platelet-rich plasma

## Abstract

**Background:**

Maxillary canine retraction following premolar extraction is a time-consuming phase of orthodontic treatment. Various adjunctive methods, including platelet concentrates, corticotomy, and piezocision, have been proposed to accelerate tooth movement; however, their relative effectiveness and safety remain unclear.

**Objective:**

To evaluate the effectiveness and safety of platelet concentrates, corticotomy, and piezocision as adjuncts to maxillary canine retraction after premolar extraction.

**Methods:**

PubMed/MEDLINE, Scopus, and Cochrane CENTRAL were searched from inception to April 30, 2026. Randomized controlled trials comparing platelet-rich plasma, injectable platelet-rich fibrin, leukocyte and platelet-rich fibrin, corticotomy, or piezocision with conventional maxillary canine retraction were included. Study selection, data extraction, and risk-of-bias assessment using RoB 2 were performed independently. Findings were synthesized narratively.

**Results:**

Twenty-five randomized studies were included. Corticotomy and piezocision generally increased canine retraction during the early postoperative period, although the effect decreased over time in several trials. Platelet concentrates produced inconsistent findings, ranging from no significant effect to small or temporary increases in movement. Evidence that these interventions shortened the complete duration of canine retraction was limited. Anchorage loss and periodontal outcomes were generally similar between groups, while findings for tipping, root resorption, pain, and adverse effects varied. Four studies had a low risk of bias, 21 raised some concerns.

**Conclusion:**

Corticotomy and piezocision may increase rate of early maxillary canine retraction, while the effects of platelet concentrates are less consistent. Current evidence does not establish a reliable reduction in total retraction duration or support a clear ranking of the interventions. Better standardized randomized trials are required.

**Systematic Review Registration:**

identifier CRD420261287411.

## Introduction

The duration of orthodontic treatment is influenced by several factors, including the severity of the malocclusion, the treatment mechanics employed, the biological response to orthodontic forces, and patient compliance. Prolonged treatment duration has been associated with an increased risk of adverse effects, including plaque accumulation, white spot lesions, periodontal deterioration, and external apical root resorption (1). Therefore, strategies aimed at reducing treatment time while maintaining effective anchorage control, periodontal health, and precise tooth movement are of considerable clinical interest. In this context, accelerated orthodontic techniques have emerged as a promising approach to enhance treatment efficiency without compromising treatment outcomes.

Premolar extraction followed by maxillary canine retraction is a routine component of orthodontic treatment for the correction of dental crowding and bimaxillary proclination. However, canine retraction often requires several months to complete, as tooth movement must occur across the extraction space and is limited by the biologic processes of periodontal ligament remodeling and alveolar bone turnover. To overcome these limitations and reduce overall treatment duration, various accelerated orthodontic approaches have been developed. These strategies aim to enhance the biological response underlying tooth movement and include surgical interventions, physical stimulation techniques, pharmacological agents, and locally administered biological mediators (2). These approaches differ in their underlying biological mechanisms, clinical applicability, and efficacy in enhancing the rate of orthodontic tooth movement.

Corticotomy and piezocision are among the most extensively investigated surgical approaches for accelerating orthodontic tooth movement. Corticotomy involves the creation of controlled cortical bone injuries, typically following flap elevation, whereas piezocision utilizes minimally invasive, flapless cortical incisions. Both techniques are believed to enhance tooth movement by triggering the Regional Acceleratory Phenomenon (RAP), a localized biological response characterized by a transient increase in bone remodeling and turnover following surgical injury, as originally described by Frost (3). This heightened remodeling activity, accompanied by a temporary reduction in bone density, facilitates more rapid tooth movement under orthodontic forces. Piezocision was subsequently introduced as a less invasive alternative to conventional corticotomy, aiming to achieve comparable biological effects while minimizing surgical trauma, patient discomfort, and postoperative morbidity (4).

Randomized trials have reported inconsistent findings regarding the effect of corticotomy and piezocision on maxillary canine retraction. While several studies have demonstrated enhanced tooth movement during the early postoperative phase, others have reported minimal or no significant acceleration compared with conventional retraction methods (5–8). Furthermore, the longevity of the acceleratory effect and its impact on the overall duration of canine retraction remain unclear.

Biological adjuncts such as platelet-rich plasma (PRP) and injectable platelet-rich fibrin (i-PRF) have been locally applied to enhance maxillary canine retraction (9–12). Similarly, leukocyte- and platelet-rich fibrin (L-PRF) has been investigated following its placement within premolar extraction sockets (13). However, meaningful comparisons among these studies are challenging because of considerable variations in preparation protocols, dosage regimens, application frequency, orthodontic mechanics, follow-up duration, and methods used to assess treatment outcomes.

Previous reviews have assessed corticotomy and piezocision during canine retraction, but the evidence is limited by small sample sizes and heterogeneity in surgical protocols (14). Platelet concentrates have generally been evaluated separately from surgical acceleration techniques, and different preparations have often been grouped despite important procedural differences. As a result, the comparative effectiveness of these interventions remains unclear. It is also uncertain whether the initial acceleration of canine retraction is sustained over time, reduces overall retraction duration, or affects anchorage, root resorption, periodontal health, pain, or other adverse outcomes.

The present systematic review aimed to evaluate randomized controlled trials investigating platelet concentrates, corticotomy, and piezocision as adjunctive interventions for maxillary canine retraction following premolar extraction. Their effectiveness was compared with conventional orthodontic retraction with respect to the rate of canine retraction and overall retraction duration. Secondary outcomes, including anchorage loss, root resorption, periodontal health, pain, and other reported adverse effects, were also assessed.

## Methodology

The present systematic review was conducted to evaluate the effectiveness of platelet concentrates, corticotomy, and piezocision in accelerating maxillary canine retraction following premolar extraction. The review was planned and reported in accordance with the Preferred Reporting Items for Systematic Reviews and Meta-Analyses 2020 guidelines (15).

A protocol was prepared before completion of study selection and data extraction and was submitted to the International Prospective Register of Systematic Reviews. The registration number for the published Prospero record is CRD420261287411.

The review question was structured according to the PICOS format:

**Population (P):** Adolescents and adults undergoing maxillary canine retraction after premolar extraction using fixed orthodontic appliances.

**Intervention (I):** Platelet concentrates, including platelet-rich plasma, injectable platelet-rich fibrin, and leukocyte and platelet-rich fibrin; corticotomy-assisted orthodontics; or piezocision-assisted orthodontics.

**Comparator (C):** Conventional maxillary canine retraction without a biological or surgical acceleration procedure.

### Outcomes (O)

**Primary Outcomes:** Rate of maxillary canine retraction and total duration of canine retraction.

**Secondary Outcomes:** Anchorage loss, canine tipping and rotation, root resorption, periodontal parameters, pain, swelling, discomfort, patient-reported outcomes, and intervention-related adverse effects.

**Study design (S):** Randomized controlled trials (RCTs) (parallel or split mouth).

### Literature search

A comprehensive electronic literature search was conducted in PubMed/MEDLINE, Scopus, and the Cochrane Central Register of Controlled Trials (CENTRAL). These databases were selected based on availability through the institutional library and their relevance to biomedical, dental, and controlled-trial literature. Searches were conducted between May 5 and May 8, 2026. The review considered literature published up to April 30, 2026. The search strategy combined controlled vocabulary terms (MeSH terms where applicable) and free-text keywords related to orthodontic canine retraction and accelerated orthodontic interventions. Search terms included combinations of: “canine retraction”, “maxillary canine movement”, “orthodontic tooth movement”, “accelerated orthodontics”, “platelet-rich plasma” OR “PRP”, “platelet-rich fibrin” OR “PRF”, “injectable platelet-rich fibrin” OR “i-PRF”, “leukocyte platelet-rich fibrin” OR “L-PRF”, “corticotomy”, “corticotomy-assisted orthodontics”, “piezocision”, and “randomized controlled trial”. (Table 1).

**Table 1:** Search strategy and search terms used.

Database	Search query	Results
PubMed	((canine retraction OR maxillary canine retraction OR orthodontic space closure) AND (platelet-rich plasma OR platelet-rich fibrin OR injectable platelet-rich fibrin OR leukocyte platelet-rich fibrin OR corticotomy OR piezocision) AND (randomised OR randomized OR clinical trial))	291
Scopus	TITLE-ABS-KEY((“canine retraction” OR “orthodontic space closure” OR “maxillary canine”) AND (“platelet-rich plasma” OR “platelet-rich fibrin” OR “injectable platelet-rich fibrin” OR “leukocyte platelet-rich fibrin” OR corticotomy OR piezocision) AND (randomised OR randomized OR “clinical trial”))	447
Cochrane	((canine retraction OR orthodontic space closure) AND (platelet-rich plasma OR platelet-rich fibrin OR injectable platelet-rich fibrin OR L-PRF OR corticotomy OR piezocision))	279
Total	Records identified across all searched databases before deduplication	1,017

Boolean operators (AND, OR) and database-specific filters were applied as appropriate. The search strategy was adapted for each database according to its indexing system and search requirements. Additionally, the reference lists of all included studies and relevant review articles were manually screened to identify potentially eligible studies that may have been missed during the electronic search.

### Inclusion and exclusion criteria for study selection

Inclusion Criteria:

- Randomized controlled trials (parallel or split-mouth) involving adolescents or adults undergoing maxillary canine retraction after premolar extraction with fixed orthodontic appliances.
- Studies evaluating PRP, i-PRF, L-PRF, corticotomy, or piezocision compared with conventional canine retraction.
- Studies reporting outcomes related to the rate, amount, or duration of canine retraction with extractable data.
- Full-text articles published in English.

Exclusion Criteria:

- Studies evaluating other orthodontic movements or acceleration techniques without separate canine retraction data.
- Studies using clear aligners, removable appliances, orthognathic surgery, distraction osteogenesis, or alveolar distraction.
- Participants with craniofacial anomalies, active periodontal disease, systemic conditions, or medications affecting bone metabolism.
- Studies lacking a conventional control group, relevant outcome data, or a randomized design.
- Studies using quasi-randomized or otherwise predictable allocation methods, such as odd/even patient numbering.
- Case reports, reviews, conference abstracts, animal/laboratory studies, and non-English or non-full-text publications.

### Study selection

Titles and abstracts were screened independently by two reviewers (A.K.V and A.S) against the predefined eligibility criteria. Full texts of potentially eligible studies were then assessed independently by the same reviewers. Disagreements at either stage were resolved through discussion and, when required, consultation with additional reviewers (A.M and M.S.R).

### Data extraction

The following information was collected and tabulated:

- Author and year
- Country
- Study design
- Randomization/allocation method
- Sample size
- Participants age
- Malocclusion/extraction pattern
- Arch/tooth movement
- Intervention category
- Comparator
- Follow up
- Outcomes reported

Data were extracted using a standardized Microsoft Excel sheet by two independent reviewers. Extracted information included study characteristics, randomization and allocation procedures, participant demographics, malocclusion and extraction patterns, arch/tooth movement, intervention category, comparators, follow-up, and outcomes reported. The detailed characteristics of each of the included studies are explained in Table 2. Discrepancies were resolved through discussion and, when necessary, consultation with third and fourth reviewers (A.M. and M.S.R.). S.K. Cross-checked the extracted data. No duplicate or overlapping publications were identified among the included studies.

**Table 2:** Study characteristics of included studies.

Study	Country	Study design/randomization	Sample	Intervention	Comparator	Follow-up	Main outcome
Alfailany et al. (7)	Syria	Parallel RCT; computer-generated sequence; opaque envelopes	*n* = 51	Corticotomy	Conventional control	To completion	Canine retraction
Eid and El-Kalza (27)	Egypt	RCT with split-mouth component; computer-generated allocation	*n* = 30	Piezocision	Contralateral control	12 weeks	Canine retraction
Abbas et al. (5)	Egypt	Split-mouth RCT; coin toss	*n* = 20	Corticotomy; piezocision	Contralateral control	3 months	Canine retraction
Raj et al. (28)	India	Split-mouth RCT; computer-generated allocation	*n* = 20	Piezocision	Contralateral control	90 days	Canine retraction
Yıldırım et al. (29)	Turkey	Split-mouth RCT; computer-generated sequence; sealed envelopes	*n* = 15	Piezocision	Contralateral control	28 days	Canine retraction
Fernandes et al. (8)	Brazil	Randomized trial with split-mouth comparisons; software-generated allocation	*n* = 47	Corticotomy; piezocision	Contralateral control	24 weeks	Canine retraction
Moradinejad et al. (30)	Iran	Split-mouth RCT; restricted randomization; coin toss for side	*n* = 32	Piezocision	Contralateral control	9 weeks	Canine retraction; anchorage loss
Al-A’athal et al. (31)	Jordan	Split-mouth RCT; permuted blocks; opaque envelopes	*n* = 23	Piezocision	Contralateral control	12 weeks	Canine retraction; anchorage loss
Syal et al. (32)	India	Prospective randomized split-mouth study; exact side sequence not reported	*n* = 12	Piezocision	Contralateral control	3 months	Canine retraction
Angel et al. (10)	India	Split-mouth RCT; computer-generated sequence; opaque envelopes	*n* = 10	PRP	Contralateral control	60 days	Canine retraction
El Timamy et al. (9)	Egypt	Split-mouth RCT; computer-generated sequence; opaque envelopes	*n* = 15	PRP	Contralateral placebo control	4 months	Canine retraction
Raza et al. (22)	India	Split-mouth RCT; computer-generated sequence; opaque envelopes	*n* = 10	Corticotomy	Contralateral control	To completion	Canine retraction; duration
Gupta et al. (18)	India	Split-mouth RCT; block randomization; opaque envelopes	*n* = 35	L-PRF	Contralateral control	4 months/to completion	Canine movement; duration
Alfawal et al. (6)	Syria	RCT with split-mouth control; computer-generated sequence; opaque envelopes	*n* = 34	Piezocision	Contralateral control	4 months	Canine retraction; duration
Aboul-Ela et al. (23)	Egypt	Split-mouth randomized study; coin toss	*n* = 13	Corticotomy	Contralateral control	4 months	Canine retraction
Zeitounlouian et al. (12)	Syria	Split-mouth RCT; computer-generated side allocation	*n* = 21	i-PRF	Contralateral control	5 months	Canine retraction
Arora et al. (19)	India	Split-mouth randomized study; opaque-envelope side allocation	*n* = 14	PRP	Contralateral control	4 months	Canine retraction
Krishna et al. (13)	India	Split-mouth RCT; computer-generated side allocation	*n* = 16 analyzed	L-PRF	Contralateral control	8 weeks	Canine retraction
Jahanbakhshi et al. (24)	Iran	Split-mouth randomized trial; sequence method not reported	*n* = 15	Corticotomy	Contralateral control	≈4 months	Canine retraction
Al-Naoum et al. (25)	Syria	Split-mouth RCT; sealed-envelope side allocation	*n* = 30	Corticotomy	Contralateral control	12 weeks	Canine retraction
Naji et al. (11)	Syria	Parallel RCT with split-mouth control; sealed-envelope allocation	*n* = 40	PRP; i-PRF	Contralateral placebo control	12 weeks	Canine retraction
Sharma et al. (26)	India	Split-mouth RCT; randomization stated; sequence method not reported	*n* = 14 analyzed	Corticotomy	Contralateral control	To completion	Canine retraction; anchorage
Gupta et al. (20)	India	Split-mouth RCT; drawing lots	*n* = 16	L-PRF	Contralateral control	5 months	Canine retraction
Pradhan et al. (33)	India	Prospective split-mouth randomized study; independent side allocation	*n* = 15	Piezocision	Contralateral control	To completion	Retraction duration; root resorption
El Gazzar et al. (21)	Egypt	Split-mouth RCT; drawing lots	*n* = 20 reported	PRP	Contralateral control	4 months	Canine retraction

Quantitative synthesis was considered within individual intervention groups and at comparable follow-up points. However, differences in intervention protocols, orthodontic mechanics, outcome assessment, and follow-up limited comparability, and paired variance data were not consistently available from the predominantly split-mouth trials. Therefore, the findings were synthesized narratively.

### Risk of bias assessment

Risk of bias was assessed independently by two reviewers (A.K.V) and (A.S) using the revised Cochrane Risk of Bias tool for randomized trials (RoB 2) (16, 17), with disagreements resolved through discussion or consultation with third and fourth reviewers (A.M. and M.S.R.). N.P.S. Cross-checked the risk of bias assessment. The assessment focused on the effect of assignment to the intervention on maxillary canine retraction and evaluated five domains: bias arising from the randomization process, deviations from intended interventions, missing outcome data, outcome measurement, and selection of the reported result. Each domain was rated as low risk of bias, some concerns, or high risk of bias, and an overall judgment was determined according to the RoB 2 algorithm. For split-mouth trials, assessments additionally considered randomization and concealment of side allocation, blinding of outcome assessment, completeness of paired observations, and the appropriateness of statistical analyses for the paired design (Figure 1).

**Figure 1:**
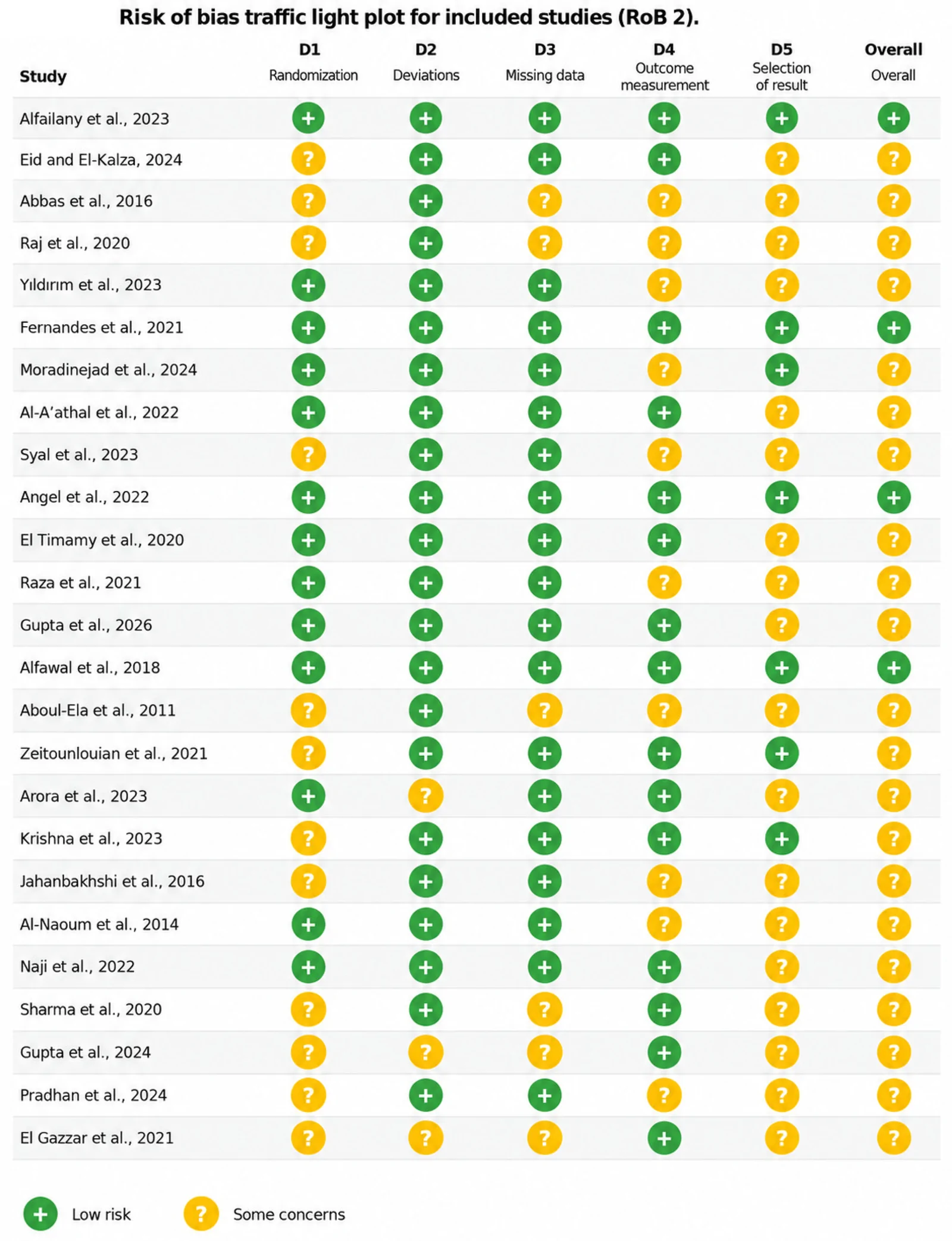
Risk of bias traffic light plot for included studies (RoB 2).

The certainty of evidence for the primary and key secondary outcomes was assessed using the GRADE approach (16). (Table 3) Evidence was evaluated for risk of bias, inconsistency, indirectness, imprecision, and publication bias, and certainty was rated as high, moderate, low, or very low. As meta-analysis was not performed, judgments regarding inconsistency and imprecision were based on the direction and consistency of findings, sample sizes, and precision of the reported estimates.

**Table 4:** Certainty of evidence for the main outcomes using the GRADE approach.

Outcome/intervention	Summary of findings	Main reasons for downgrading	Certainty
Canine retraction rate/amount—PRP	Variable early effect; benefit was absent or not sustained in some studies	Risk of bias, inconsistency, imprecision	Very low
Canine retraction rate/amount—i-PRF	No consistent sustained increase in retraction	Risk of bias, imprecision	Low
Canine retraction rate/amount—L-PRF	Generally greater early movement	Risk of bias, imprecision	Low
Canine retraction rate/amount—corticotomy	Generally greater early movement; effect decreased with time	Risk of bias, imprecision	Low
Canine retraction rate/amount—piezocision	Greater early movement in most studies, with some null findings	Risk of bias, imprecision	Low
Total canine retraction duration	Evidence was limited; some studies reported shorter duration with L-PRF, corticotomy, or piezocision	Risk of bias, sparse data, imprecision	Low to very low
Anchorage loss	No consistent difference between intervention and control groups	Risk of bias, imprecision	Low to very low
Root resorption	Findings were limited and not fully consistent across interventions	Risk of bias, inconsistency, imprecision	Low to very low
Pain/adverse effects	Effects were mainly transient; adverse-event reporting was limited	Risk of bias, imprecision, limited reporting	Low to very low

## Results

A total of 1,017 records were identified through database searches. After the removal of 336 duplicates, 681 records were screened, resulting in 42 reports sought for retrieval. Three reports could not be obtained, and 39 full-text articles were assessed for eligibility. Following exclusion of 14 studies, 25 randomized controlled trials were included in the qualitative synthesis (Figure 2). The included studies, published between 2011 and 2026, predominantly used a split-mouth design and investigated maxillary canine retraction following bilateral premolar extraction. Interventions evaluated included platelet concentrates (9 studies), corticotomy (8 studies), and piezocision (10 studies), with some multi-arm trials contributing to more than one category (5–13, 18–33). Sample sizes ranged from 10 to 51 participants, with considerable variation in intervention protocols, orthodontic mechanics, anchorage methods, follow-up periods, and outcome assessment techniques.

**Figure 2:**
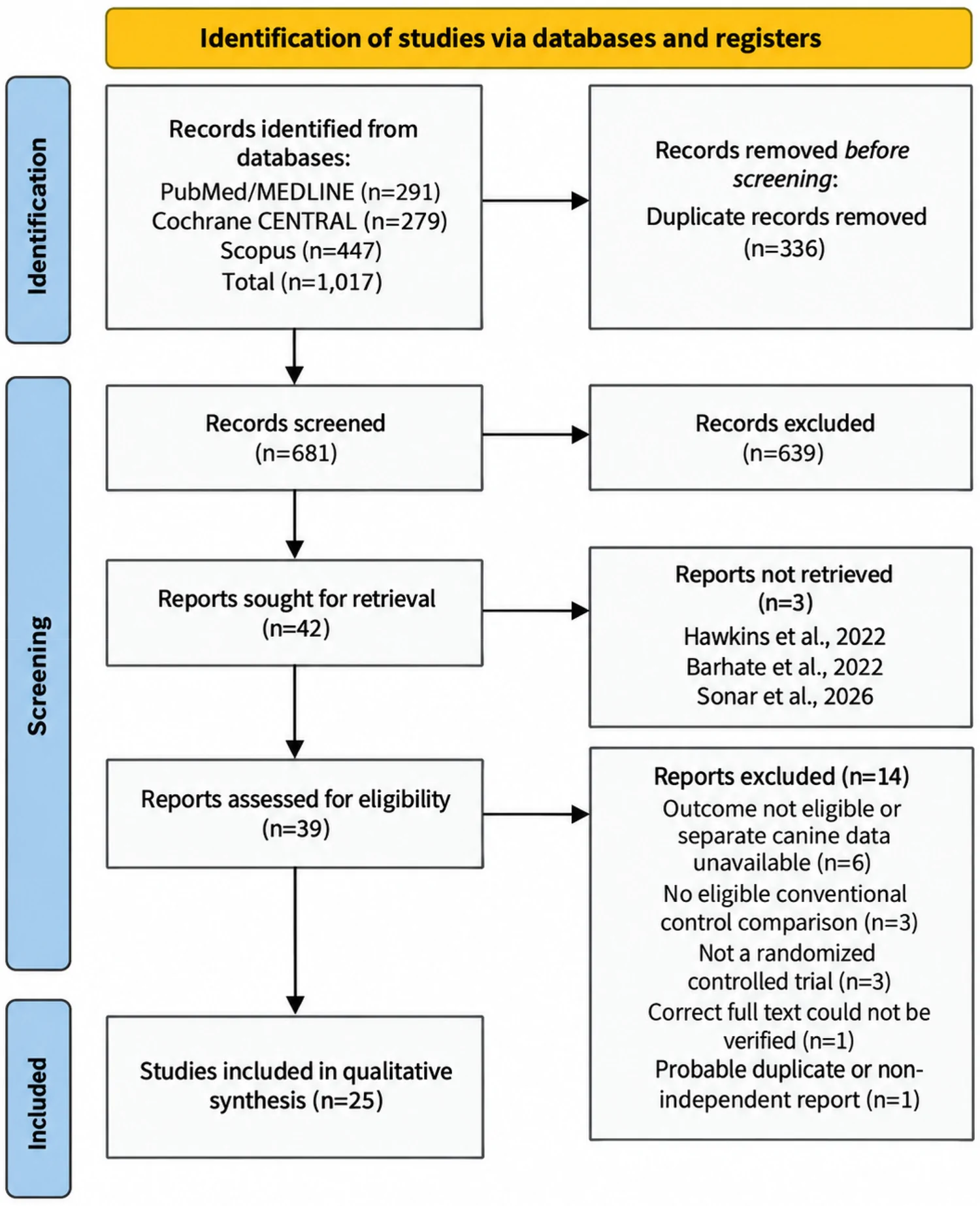
PRISMA 2020 flow diagram: 1,017 records; 336 duplicates removed; 681 screened; 39 full texts assessed; 14 excluded; 25 RCTs included.

Regarding canine retraction, platelet concentrates demonstrated variable results. PRP showed accelerated movement in some studies but no significant or sustained benefit in others (9–11, 19, 21). The numerical primary outcomes reported by individual studies are summarized in Table 4. Similarly, i-PRF did not show a consistent sustained acceleratory effect, with one study reporting a significant difference during a single follow-up interval and another finding no significant overall benefit (11, 12). L-PRF generally enhanced early canine retraction, although the effect was often modest and diminished over time (13, 18, 20). Corticotomy was associated with increased canine retraction during the early postoperative period in most studies (5, 7, 22–26), although the effect tended to decrease with time, and one study found no significant advantage over conventional retraction (8). One study reported a reduction in overall canine retraction duration following corticotomy (22). Likewise, piezocision accelerated canine retraction during the initial weeks or months in most trials (5, 6, 27–30, 32, 33), but the benefit generally lessened over time. Two studies reported no significant acceleratory effect (8, 31), and repeated piezocision did not provide additional benefit compared with a single procedure (27). Across the categories, acceleration was observed during the early stages of retraction, whereas there was limited evidence for a reduction in the total duration of canine retraction. The primary numerical outcomes reported by the individual studies are summarized in Table 4.

**Table 3:** Primary numerical outcomes of the included studies.

Study	Intervention	Follow-up	Key numerical result (intervention vs. control)	Total retraction duration	Main finding
Alfailany et al. (7)	Corticotomy	To completion	M1: 2.79 ± 0.39 vs. 1.12 ± 0.16 mm	NR	Greater movement during the first 2 months
Eid and El-Kalza (27)	Piezocision	12 weeks	Single: 4.49 ± 0.34 vs. 2.77 ± 0.29 mm; multiple: 4.68 ± 0.27 vs. 2.79 ± 0.29 mm	NR	Both protocols increased retraction
Abbas et al. (5)	Corticotomy; piezocision	3 months	T6: corticotomy 1.22 ± 0.08 vs. 0.58 ± 0.04 mm; piezocision 0.99 ± 0.10 vs. 0.60 ± 0.04 mm	NR	Both increased movement; corticotomy generally greater
Raj et al. (28)	Piezocision	90 days	4.25 ± 0.60 vs. 2.88 ± 0.40 mm at 90 days	NR	Greater movement at all assessed intervals
Yıldırım et al. (29)	Piezocision	28 days	1.27 ± 0.36 vs. 0.70 ± 0.25 mm at 28 days	NR	Greater early distalization
Fernandes et al. (8)	Corticotomy; piezocision	24 weeks	Corticotomy: 3.83 ± 1.00 vs. 3.97 ± 1.41 mm; piezocision: 2.93 ± 1.11 vs. 4.05 ± 0.96 mm	NR	No corticotomy benefit; piezocision lower than control
Moradinejad et al. (30)	Piezocision	9 weeks	3.56 ± 0.05 vs. 2.42 ± 0.06 mm at 9 weeks	NR	Greater movement; effect decreased over time
Al-A’athal et al. (31)	Piezocision	12 weeks	Average facial height: 1.48 ± 0.74 vs. 1.88 ± 0.83 mm; high facial height: 1.40 ± 0.85 vs. 1.76 ± 0.62 mm	NR	No significant acceleratory effect
Syal et al. (32)	Piezocision	3 months	3.97 ± 1.54 vs. 1.78 ± 0.42 mm	NR	Greater retraction with piezocision
Angel et al. (10)	PRP	60 days	3.19 ± 0.27 vs. 2.30 ± 0.20 mm	NR	Greater movement over 2 months
El Timamy et al. (9)	PRP	4 months	Total: 4.57 ± 1.32 vs. 4.53 ± 1.12 mm	NR	Early benefit only; total movement similar
Raza et al. (22)	Corticotomy	To completion	M1: 1.36 ± 0.33 vs. 0.81 ± 0.06 mm	5.7 vs. 7.1 months	Faster early movement and shorter retraction duration
Gupta et al. (18)	L-PRF	4 months/to completion	Cusp movement: 3.53 ± 0.23 vs. 2.71 ± 0.22 mm	6.01 ± 0.53 vs. 6.77 ± 0.49 months	Greater cusp movement; greater tipping also observed
Alfawal et al. (6)	Piezocision	4 months	M1: 1.65 ± 0.40 vs. 0.83 ± 0.18 mm	≈1.17-month reduction reported	Early acceleration with diminishing effect
Aboul-Ela et al. (23)	Corticotomy	4 months	M1: 1.89 vs. 0.75 mm/month	NR	Marked early acceleration; little difference by month 4
Zeitounlouian et al. (12)	i-PRF	5 months	Total: 3.90 ± 1.36 vs. 3.94 ± 1.12 mm	NR	No sustained overall benefit
Arora et al. (19)	PRP	4 months	M1: 1.355 ± 0.699 vs. 1.265 ± 0.698 mm	NR	No significant difference at any interval
Krishna et al. (13)	L-PRF	8 weeks	2.29 ± 0.19 vs. 2.01 ± 0.30 mm	NR	Small but significant increase in movement
Jahanbakhshi et al. (24)	Corticotomy	≈4 months	Overall: 1.8 ± 0.17 vs. 1.1 mm/month	NR	Greater retraction velocity, largest early
Al-Naoum et al. (25)	Corticotomy	12 weeks	Week 1: 0.739 ± 0.365 vs. 0.201 ± 0.149 mm/week	NR	Greater velocity throughout follow-up
Naji et al. (11)	PRP; i-PRF	12 weeks	PRP 21 d: 1.95 ± 1.47 vs. 1.15 ± 1.04 mm; i-PRF total: 4.60 ± 2.26 vs. 4.88 ± 2.16 mm	NR	PRP benefit limited to first 21 days; no i-PRF benefit
Sharma et al. (26)	Corticotomy	To completion	T1: 1.276 ± 0.390 vs. 0.818 ± 0.181 mm	NR	Greater movement only during the first two intervals
Gupta et al. (20)	L-PRF	5 months	Total: 6.407 ± 0.336 vs. 5.546 ± 0.663 mm	NR	Greater total movement, mainly in first 2 months
Pradhan et al. (33)	Piezocision	To completion	Monthly movement not reported	4.66 ± 0.52 vs. 5.72 ± 0.65 months	Shorter canine distalization duration
El Gazzar et al. (21)	PRP	4 months	Total: 4.331 ± 0.163 vs. 4.274 ± 0.139 mm	NR	Statistically significant but small total difference

Secondary outcomes showed no consistent differences in anchorage loss between intervention and control groups, although some studies reported greater canine tipping on the intervention side (18, 27). Findings on root resorption were conflicting, with some corticotomy studies reporting reduced resorption, while one piezocision study found increased resorption (5, 22, 27); several others reported no significant differences (20, 26, 28, 33). Periodontal parameters were generally comparable between groups. Postoperative pain and discomfort were commonly reported during the first few days after surgery or injection but subsided with time. Apart from one reported case of bone sequestration following piezocision (8), adverse events were limited. Risk of bias assessment revealed that four studies had a low overall risk of bias and 21 raised some concerns. The certainty of evidence ranged from low to very low across the main outcomes. Certainty was limited primarily by risk-of-bias concerns, small sample sizes, inconsistent findings for some outcomes, and limited reporting of secondary outcomes. The GRADE assessment is summarized in Table 3.

## Discussion

This systematic review evaluated 25 randomized studies on platelet concentrates, corticotomy, and piezocision during maxillary canine retraction. Most interventions produced some increase in canine retraction during the early observation period. The effect was usually greatest during the first few weeks or months and tended to decrease thereafter. However, the magnitude and duration of acceleration varied across studies, and a greater monthly rate of movement did not consistently translate into a shorter total duration of canine retraction.

The findings for platelet concentrates were variable. Several PRP studies reported greater canine retraction during the early follow up period (9–11, 21). In contrast, Arora et al. found no significant difference from conventional retraction (19). El Timamy et al. reported an early increase in movement, but total movement after four months was similar between the PRP and control sides (9). The i-PRF trials did not demonstrate a consistent sustained acceleratory effect. Zeitounlouian et al. found a significant difference during only one monthly interval, while total movement after five months was almost identical between sides (12). Naji et al. did not find a significant acceleratory effect (11).

L-PRF produced greater movement in all three included trials, although the size and duration of the effect differed (13, 18, 20). Krishna et al. reported an additional 0.28 mm of movement over eight weeks (13). Gupta et al. found that the difference was concentrated within the first two months (20). The later Gupta trial reported greater canine cusp movement over four months, but this was accompanied by greater tipping (18). Greater crown movement may therefore not represent an equivalent increase in bodily tooth movement.

Variation in platelet concentrate protocols may partly account for the differences between studies. PRP and i-PRF were injected into the tissues adjacent to the canine, whereas L-PRF was placed within the extraction socket. Preparation methods, centrifugation protocols, activation, dosage, application site, and frequency of administration also differed. These preparations should therefore not be considered as a single uniform intervention. Such variability in preparation and application protocols may have influenced the biological response and contributed to the inconsistent findings reported across studies. Standardization of platelet concentrate protocols would improve comparability in future research.

Most corticotomy studies reported faster canine retraction during the early postoperative period (5, 7, 22–26). Aboul-Ela et al. found the greatest difference during the first two months, with a smaller difference during the third and fourth months (23). Sharma et al. similarly reported significant acceleration during the first two 3-week intervals only (26). Alfailany et al. observed greater movement during the first two months, but the effect was not maintained during the later stages or at completion of retraction (7). Fernandes et al., however, found no significant increase in cumulative canine retraction after corticotomy (8).

Only a limited number of corticotomy studies reported the total duration of canine retraction. Raza et al. found that completion required 5.7 months on the corticotomy side and 7.1 months on the control side (22). Other trials mainly reported movement over fixed intervals. Their findings therefore show an increase in the early rate of movement but do not establish that the full retraction phase was consistently shortened.

Most piezocision studies also reported greater movement during the early observation period (5, 6, 27–30, 32, 33). Alfawal et al. found the largest effect during the first two months and reported a reduction of approximately 1.17 months in retraction duration (6). Pradhan et al. also reported a shorter duration on the piezocision side (33). Eid and El-Kalza found that both single and repeated piezocision increased movement, but repeated application did not provide a significant additional benefit (27).

Not all studies favoured piezocision. Al-A’athal et al. found no significant increase in either facial height group (31). Fernandes et al. reported lower cumulative canine retraction on the piezocision side than on the control side (8). Differences in the number, depth, and position of cortical incisions, timing of force application, anchorage, and outcome measurement may have contributed to the variation in findings. Variation in surgical technique is also likely to influence the regional acceleratory phenomenon, making direct comparison between studies difficult.

Anchorage loss was assessed in several studies, but no consistent difference was found between intervention and control groups (5–8, 11, 12, 20, 23, 26, 30, 31). Canine tipping and rotation were not consistently reduced. Eid and El-Kalza reported greater tipping after piezocision (27), while Gupta et al. found greater cusp movement together with greater tipping after L-PRF placement (18). These findings indicate that acceleration of crown movement does not necessarily improve the quality of tooth movement.

The findings for root resorption were also mixed. Abbas et al. and Raza et al. reported less resorption on corticotomy sides (5, 22). Eid and El-Kalza found higher resorption scores after piezocision (27). Other studies reported no significant difference between intervention and control groups (20, 26, 28, 33). The imaging methods and follow-up periods varied, which limited comparison between studies.

Periodontal outcomes were generally similar between intervention and control sides. Most trials did not report clinically important differences in probing depth, attachment level, gingival recession, or plaque-related indices (5, 23, 26, 28). The follow-up periods were relatively short, and long-term periodontal effects were not assessed.

Pain and discomfort were mainly confined to the first few days after intervention. Corticotomy was associated with transient postoperative pain, swelling, or discomfort (22, 25, 26). Piezocision was generally associated with less morbidity, although Fernandes et al. reported one case of bone sequestration (8). PRP injections caused short-term pain in some studies but were not associated with serious complications (9, 10, 21). No major adverse effects were reported after L-PRF placement (13, 18).

The overall risk of bias should be considered when interpreting these findings. Four studies were judged to have a low risk of bias, while the remaining 21 raised some concerns. The most frequent concerns were unclear allocation, absence or retrospective registration of a trial protocol, incomplete reporting of blinding, and uncertainty regarding analyses. Three split-mouth studies used independent-samples tests for intervention–control comparisons (20, 32, 33), while one study reported inconsistent statistical methods (24). These findings were interpreted cautiously, as paired re-analysis was not possible from the reported data. The certainty of evidence was generally low to very low, indicating limited confidence in the magnitude and consistency of the observed effects.

Most studies had small sample sizes. Although split-mouth designs reduce variability between participants, they require appropriate paired analysis (16). In addition, many studies measured crown movement using a single dental landmark. This may overestimate acceleration when greater movement occurs through tipping rather than bodily distalization.

The main strength of this review was its restriction to randomized studies evaluating the same clinical phase of treatment. Platelet concentrates, corticotomy, and piezocision were considered together, and movement rate was assessed alongside duration, anchorage loss, root resorption, periodontal outcomes, pain, and adverse effects.

The available evidence suggests that corticotomy and piezocision can increase maxillary canine retraction during the early postoperative period. Platelet concentrates may also produce an early increase, but their effects were less consistent. None of the three approaches showed a uniform benefit across all studies, and the evidence does not support a reliable ranking of the interventions.

The findings of this review should be interpreted within the characteristics of the included studies. Most trials enrolled healthy adolescents or young adults undergoing bilateral maxillary first premolar extraction and used split-mouth designs. Therefore, the results are most applicable to patients with similar clinical characteristics, while their generalizability to other patient groups and treatment protocols remains uncertain.

## Limitations

Meta-analysis was not performed because of clinical and methodological heterogeneity across intervention protocols, orthodontic mechanics, follow-up intervals, and outcome assessment methods, together with inconsistent reporting of paired variance data in the predominantly split-mouth trials. This also limited direct comparison between studies. Most trials included small sample sizes, and many raised concerns regarding risk of bias. The duration for canine retraction was reported less frequently than movement over fixed intervals, making it difficult to determine whether early acceleration resulted in a meaningful reduction in overall treatment time. Differences in force application, anchorage systems, and methods used to assess canine retraction further limited direct comparison across studies. Follow-up periods were generally short, limiting assessment of long-term outcomes such as treatment stability, periodontal health, and root resorption. Publication bias could not be formally assessed because fewer than 10 studies contributed to individual intervention–outcome comparisons. The predominance of small, single-center trials also means that small-study effects cannot be excluded. These limitations should be considered when interpreting the findings of this review.

## Clinical implications & future scope

For clinical practice, corticotomy and piezocision may accelerate the initial phase of maxillary canine retraction. However, their benefits appear greatest during the early postoperative period, and current evidence does not establish a consistent reduction in the complete duration of canine retraction. In contrast, platelet concentrates such as PRP, i-PRF, and L-PRF have shown inconsistent effectiveness and cannot currently be recommended as reliable standalone acceleration methods. Overall, no consistent differences in periodontal health or anchorage loss were reported between intervention and control groups, although these outcomes were not uniformly assessed across studies.

Future randomized controlled trials should adopt standardized platelet preparation protocols, surgical techniques, orthodontic protocols, and outcome assessment methods to improve comparability between studies. Adequately powered multicenter trials with longer follow-up are needed to determine whether early acceleration results in meaningful reductions in canine retraction duration without compromising periodontal health, root resorption, or long-term stability. Future studies should also evaluate patient-reported outcomes, cost-effectiveness, and patient-specific factors that may influence treatment response to support more predictable and individualized orthodontic care.

## Conclusion

Corticotomy and piezocision may increase maxillary canine retraction during the early stages of retraction, whereas the effects of platelet concentrates are less consistent. Evidence that these interventions shorten the complete duration of canine retraction remains limited. No clear superiority among the techniques could be established because of variation in protocols, outcome measurement, and risk of bias.

## Data Availability

The original contributions presented in the study are included in the article/Supplementary Material, further inquiries can be directed to the corresponding author.
